# Type of Milk Feeding and Introduction to Complementary Foods in Relation to Infant Sleep: A Systematic Review

**DOI:** 10.3390/nu13114105

**Published:** 2021-11-16

**Authors:** Xiaoxi Fu, Amy L. Lovell, Andrea J. Braakhuis, Richard F. Mithen, Clare R. Wall

**Affiliations:** 1 Department of Nutrition and Dietetics, Faculty of Medical and Health Sciences, University of Auckland, Auckland 1142, New Zealand; xiaoxi.fu@auckland.ac.nz (X.F.); a.lovell@auckland.ac.nz (A.L.L.); a.braakhuis@auckland.ac.nz (A.J.B.); 2 The Liggins Institute, University of Auckland, Auckland 1142, New Zealand; r.mithen@auckland.ac.nz

**Keywords:** infant, 12 months and under, infant feeding mode, sleep, complementary feeding, night wakings, sleep duration, night-time sleep, systematic review

## Abstract

Inconsistent conclusions from infant sleep and feeding studies may influence parents feeding-related decisions. This study aimed to systematically review the existing literature on infant sleep and its relation to the timing of introduction to complementary foods and type of milk feeding to better understand their role(s) in infant sleep. Cohort, longitudinal, cross-sectional studies, and controlled trials were identified using online searches of five databases up to April 2020. Twenty-one articles with a total of 6225 infants under 12 months-of-age were eligible. Exclusively breastfed infants (≤6 months-of-age) had a greater number of night wakings, but most studies (67%) reported no difference in night-time and 24 h sleep duration compared to formula-fed infants. However, after 6 months-of-age, most studies (>65%) reported breastfed infants to sleep less in the night-time and over 24 h compared to formula-fed infants. Furthermore, studies reported no association between the timing of introduction to complementary foods and infant sleep duration (<12 months-of-age). Future studies using standardized methodologies and definitions, transdisciplinary expertise, and longitudinal design are required to better understand the complex role of feeding on sleep.

## 1. Introduction

Feeding type and sleep patterns are dynamic processes throughout the first year of life and have significant effects on health and development [1,2]. The World Health Organization (WHO) recommends exclusive breastfeeding for the first completed six months of life, with the introduction of complementary foods after six months-of-age [2]. Breastfeeding provides short- and long-term benefits to both infants and mothers, including protection against acute and chronic disorders among infants and as they grow older [3,4]. Sleep during the first year of life is especially important due to the rapid changes that occur in the consolidation of sleep/wake patterns [5,6,7]. The quantity and quality of an infant’s sleep are associated with cognitive function such as the development of memory and language [8], and the ability to learn [9,10]. In addition, insufficient sleep and sleep problems have been associated with later obesity [11] and behavioral issues such as tantrums and other behavioral management problems [12]. Frequent and extended night wakings, one of the most common infant-sleep-related problems, has also been shown to affect infant health and development [13,14,15]. Therefore, sufficient sleep during infancy is a priority [8] and is often one of the main issues reported by new parents, with frequent parental night wakings shown to affect parent mood and function [13,14,15,16]. An estimated 20–30% of children experience sleep problems during the first three years of life according to a cross-sectional study conducted in New Zealand and Australia, with one-third of parents reporting their infants as having a sleep problem [17]. 

A number of studies have examined the relationship between sleep and feeding among infants. The timing of introduction to complementary foods has been associated with infant sleep patterns, with breastfeeding reportedly playing a role in increasing sleep disturbances [17,18,19,20,21], while other studies have not found such significance [22,23]. The potential association between type of milk feeding or the timing of introduction to complementary foods and sleep may drive parental beliefs that early introduction to complementary foods or changes to the type of milk feeding, contrary to current recommendations [2], may improve their infants’ sleep patterns [19,24]. The lack of consistency of the available evidence could be a source of confusion for parents, thereby affecting feeding-related decisions during the first year of life. 

No systematic review has been completed to determine any associations between the timing of introduction to complementary foods and type of milk feeding on sleep in infants 12 months-of-age and younger. Therefore, this review aimed to systematically evaluate the existing literature to increase our understanding of this topic. 

## 2. Materials and Methods

**Figure 1:**
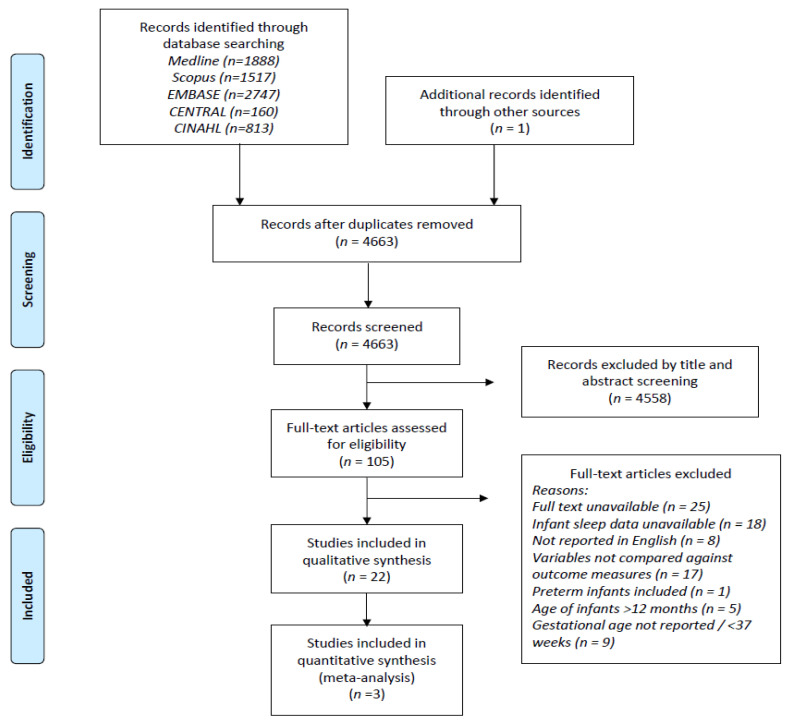
Preferred Reporting Items for Systematic Reviews and Meta-Analyses (PRIMSA) flow chart. The PRISMA flow chart is used for identification and selection of articles to be included for systematic review. A total of 7125 abstracts were retrieved using the search strategy. After removal of duplications, 4663 were screened by title and abstract, leaving 105 studies eligible for full-text review. Among the eligible studies, 84 articles were excluded due to the reasons listed. The remaining 21 studies were included for qualitative synthesis.

The review was registered with PROSPERO, the International Prospective Register of Systematic Reviews (Ref: CRD42020172830), which documents the inclusion and exclusion criteria for the review. Study selection and data were collected according to the 2009 Preferred Reporting Items for Systematic Reviews and Meta-Analyses (PRISMA) guidelines [25] and are displayed in Figure 1.

### 2.1. Search Strategy

A search strategy (Supplementary Material, Table S1) was developed to identify studies that measured sleep, with the timing of introduction to complementary foods or type of milk feeding in infants 12 months-of-age and under. A preliminary search was conducted in March 2020, with the final search completed in five electronic databases (Ovid Medline, Ovid Embase, Scopus, Cochrane library (CENTRAL), and CINAHL) in April 2020. Medical Subject Heading (MeSH) terms and free terms were used for all databases, and a combination of subject headings and keywords were used for Ovid Medline and Embase. Only studies in English were included, and publication date restriction was not applied. Hand searches and independent reviews of reference lists were carried out to explore potentially eligible studies. 

Studies included infants aged 12 months and younger, healthy, born at term (≥37 weeks gestation), measured infant sleep, and analyzed infant sleep data in relation to breastfeeding (and/or infant formula) or introduction to complementary foods, or both. Studies were excluded if infants were preterm (<37 weeks), did not include any breastfeeding infants, investigated sleep by disease state, or if effects of a particular product or products were tested against infant sleep. Reviews (systematic, meta-analyses, and editorials without original data), qualitative studies, case studies, and animal studies were excluded.

### 2.2. Selection of Studies 

Citations of the searched results from all five databases were downloaded into Endnote X9 citation management software (Clarivate Analytics, Philadelphia, USA) [26] and duplicates removed. One reviewer (X.F.) screened the study titles and abstracts using Endnote. Only studies that clearly did not meet the inclusion criteria were excluded during this stage, and all potentially relevant studies were retrieved for full-text review, which was carried out independently by X.F. and A.L.L. Screenings were based on predefined inclusion and exclusion criteria.

### 2.3. Data Extraction and Quality Assessment

The extraction form was developed by X.F. using examples in the relevant literature. The form was independently piloted by two reviewers (X.F. and A.L.L.) using the first five included studies. Adjustments were made, and the finalized form was used by X.F. and A.L.L. for independent data extraction on all included studies (*n =* 21). In the case of unresolved discrepancies, third (A.J.B.) and fourth (C.R.W.) reviewers were involved as the final decision makers. Relevant outcome data extracted included the mean, standard deviation or other variance data, and participant numbers in both control and intervention groups. Missing data were requested from corresponding authors via email. If no response was obtained from the authors, studies were excluded from the qualitative synthesis. Sleep data extracted included 24 h sleep duration, total night-time sleep, night-waking frequency, duration of night wakings, longest sleep period, and sleep onset latency. Qualitative synthesis was carried out with the assistance of tabulation of the results by grouping extracted data with similar outcomes within each age group (≤6 months, >6 months, 0–12 months). Criteria for meta-analysis were sleep variables with at least three independent studies that used a validated sleep assessment tool with available average and variance data deemed appropriate to pool. However, due to the high heterogeneity among the included studies, pooling the results was not possible, therefore meta-analysis was not deemed appropriate. Details on the heterogeneity of the included studies and inability to conduct a meta-analysis will be discussed in the limitations.

For studies evaluating the association between infant sleep and the type of milk feeding, standardized definitions for type of feeding were developed post hoc after study selection, deviating from the original PROSPERO protocol. This decision was made by the authors due to wide variation in the methodologies employed to categorize infants based on the type of milk feeding and timing of introduction to solids, as most studies were observational in design, with a lack of a comparator or control group. The agreed post hoc feeding groups were exclusive breastfeeding, breastfeeding, and formula feeding. Infants 6 months and younger categorized into the exclusive breastfeeding group had breastmilk only, as per the WHO definition [27]. Infants categorized into the breastfeeding group were partially or predominantly fed with breastmilk, and, infants categorized into the formula feeding group were exclusively or predominantly fed with infant formula. In the studies of infants 6 months and older, the following definitions were used to indicate if the infants had been introduced to complementary foods or not. Infants in the breastfeeding group were indicated as breastfeeding +/− food as they were partially or predominantly breastfed with or without complementary foods. Infants in the formula feeding group were indicated as formula feeding +/− food, who were predominantly formula-fed with the addition of, or exclusively formula-fed without the addition of, complementary foods. For studies that examined the association between infant sleep and the timing of introduction to complementary foods, infant age was recorded as reported (i.e., ≤12 weeks vs. >12 weeks), and the above type of feeding definitions did not apply.

A quality assessment tool adapted from the Mixed Methods Appraisal Tool (MMAT, v.2018) [28] was used by two reviewers (X.F. and A.L.L.) to independently assess the quality of the included studies. The MMAT provides a checklist for describing and critically appraising included studies, providing a score that considers intra- and inter- individual variation. The questions from the checklist examined the representativeness of participants, appropriate measurements, complete outcome data, accounted confounders, and intended intervention administration. The tool was modified through the addition of the question “is the tool for measuring sleep validated or an objective assessment?”. This question assessed the quality of sleep data extracted from the included studies. Following modification, the tool was scored out of 6 points (Supplementary Table S2). In this review, a low-quality study was defined by a score of 3 points and lower. Sensitivity analysis was carried out on sleep variables by comparing results before and after removal of the low-quality studies (≤3 points).

## 3. Results

A total of 4662 studies were identified using the search results from all databases and other sources and after removal of duplicates (Figure 1). Table 1 summarizes the characteristics of all 21 studies included in the systematic review. Among these, nine (43%) were cross-sectional [19,29,30,31,32,33,34,35,36], six (29%) were cohort studies [23,37,38,39,40,41], five (24%) were longitudinal studies [17,42,43,44,45], and one study combined randomized control trials [46]. The studies were mainly conducted in the United Kingdom (*n* = 5), and the United States (*n* = 8), making up more than half of the included studies. The remainder were from Israel (*n* = 2), Canada (*n* = 1), China (*n* = 2), Japan (*n* = 1), Portugal (*n* = 1), and South Korea (*n* = 1). The sample size of the included studies ranged from 20 to 1676 participants, totaling 6225 infants under 12 months-of-age. Subjective methods including sleep dairies/timetables, Brief Infant Sleep Questionnaire (BISQ), and other sleep questionnaires were used in 15 studies [17,19,30,31,32,33,34,35,36,37,38,39,41,42,46] to assess sleep. The remaining six studies used objective methods to measure sleep, such as an actigraph or electroencephalogram (EEG). Four of these studies used a combination of subjective and objective measures, e.g., sleep diaries in conjunction with the actigraph (actimetry sensor) [40,43,44,45], one used a one channel (EEG) [29] and sleep diary, and another used EEG with a questionnaire [23] to capture infants’ sleep patterns.

### 3.1. Type of Milk Feeding and Infant Sleep

All twenty-one studies reported on type of milk feeding in relation to infant sleep patterns as shown in Table 2. The type of milk feeding was reported prospectively by parents or caregivers through questionnaires [17,19,23,30,31,35,38], interviews [32,46], feeding logs [36,37,45], and by maternal self-report [33,34,39,40,43,44] except for one study [41], that assessed type of milk feeding retrospectively through a questionnaire. Two studies [29,42] did not specify their assessment methods.

#### 3.1.1. 24 h Sleep Duration

Among infants aged 6 months and younger, exclusive breastfeeding was not associated with 24 h sleep duration in one study [44] and no difference in sleep duration compared to formula-feeding was reported in a cohort study [38]. However, one study reported significantly longer sleep duration in exclusively breastfed infants compared to formula-fed infants [32]. There was no reported difference in sleep duration of breastfed infants compared to formula-fed infants in four studies [23,29,31,38]. In contrast, one study found that breastfed infants had significantly shorter sleep duration [34], while another study reported a significantly longer sleep duration compared to formula-fed infants [37].

Among infants older than 6 months, 24 h sleep duration did not differ between breastfed and formula-fed infants in one study [23], whilst two other studies reported significantly shorter sleep duration in breastfed infants compared to formula-fed infants [42,46].

Breastfeeding was associated with significantly shorter sleep duration compared to formula feeding in a study that examined infants 0 to 8 months-of-age [39]. 

#### 3.1.2. Total Night-Time Sleep

Among infants aged 6 months and younger, no difference in total night-time sleep was reported between exclusively breastfed and formula-fed infants in one cohort and one longitudinal study, where sleep was measured at multiple time points [38,43]. However, one study reported exclusively breastfed infants at 3 and 4 months-of-age were less likely to sleep through the night compared to formula-fed infants [45]. There was no reported difference in total night-time sleep in breastfed infants compared to formula-fed infants in three studies [31,34,38]. However, three other studies reported a significantly shorter night-time sleep duration experienced by breastfed infants [29,33,41], while one study reported breastfed infants to have a significantly longer night-time sleep duration compared to formula-fed infants [37]. 

Among infants older than 6 months, breastfeeding was inversely associated with night-time sleep duration in two studies [30,41]. 

No difference in night-time sleep duration was found in breastfed infants compared to formula-fed infants aged 3 to 12 months [17] and 0 to 8 months [39]. 

#### 3.1.3. Night-Waking Frequency 

In two studies, there was no reported differences in the frequency of night wakings in infants aged 6 months and younger who were exclusively breastfed or formula-fed [38,43]. However, one of these studies reported that infants that exclusively breastfed had significantly fewer night wakings at 16 weeks compared to formula-fed infants [43]. The majority of studies (*n* = 3) reported that exclusively breastfed infants had a significantly higher number of night-time wakings compared to formula-fed infants [38,40,44]. In contrast, the majority of the studies (*n* = 5) of breastfed infants and formula-fed infants reported no association [29,34,36,37,38], with two studies reporting that breastfed infants had significantly more night-time wakings than formula-fed infants [31,33]. 

No association between night-waking frequency and breastfeeding compared to formula-fed infants older than six months was reported in one study [19]. Another study found breastfed infants to have a significantly greater frequency of night awakening compared to formula-fed infants [30].

In one study, no differences were found in night-waking frequency in infants aged 0–8 months who were breastfed or formula-fed [39]. However, breastfed infants had a significantly greater number of night-time wakings than formula-fed infants aged 3 to 12 months [17] and 2 to 12 months [35]. 

#### 3.1.4. Duration of Night Wakings 

Among infants aged 6 months and younger, no difference was reported in duration of night wakings between exclusively breastfed and formula-fed infants in one study [38]. However, another study reported a significantly longer duration of night wakings in exclusively breastfed infants compared to formula-fed infants [45]. 

Among infants older than 6 months, breastfeeding was reported to result in a significantly longer duration of night wakings compared to formula-fed infants in one study [30].

#### 3.1.5. Longest Sleep Period 

Two studies reported no difference in longest sleep period between exclusive breastfeeding and formula feeding among infants aged 6 months and younger [38,43]. In contrast, two studies [32,38] found exclusively breastfed infants to have a significantly shorter longest sleep period compared to infants who were formula-fed. Of those studies, two studies measured the longest sleep period at multiple time points, one cohort [38] and another longitudinal design [43]. The longitudinal study [43] also found exclusively breastfed infants at 18 weeks-of-age to have a significantly longer longest sleep period as compared to formula-fed infants. Breastfed infants, when compared to formula-fed infants, reported no difference in longest sleep period in one study [38], while another study reported breastfed infants were more likely to wake parents in a four hour period than formula-fed infants [36].

No difference in longest sleep period duration was found in breastfed infants compared to formula-fed infants aged 3 to 12 months [17] and 0 to 8 months [39]. 

#### 3.1.6. Sleep Onset Latency 

Among infants aged 6 months and younger, one study reported no difference in sleep onset latency between exclusively breastfed and formula-fed infants [38], while another study reported exclusive breastfeeding was associated with later sleep onset time [44]. When breastfed infants were compared to formula-fed infants, no difference in sleep onset latency was reported in one study [38], while another study reported longer sleep onset duration associated with breastfed infants [29]. 

No difference in sleep onset latency was found in breastfed infants when compared to formula-fed infants aged 3 to 12 months [17]. 

### 3.2. Introduction to Complementary Foods and Infant Sleep 

Four studies (two cohort, one combined RCT, and one cross-sectional) examined the association between the timing of introduction to complementary foods and infant sleep (Table 3). All sleep measurements were subjective, and information on timing of introduction to complementary foods was retrospectively collected from parents or caregivers.

#### 3.2.1. 24 h Sleep Duration

Three studies reported the relationship between the timing of introduction to complementary foods and 24 h sleep duration (Table 3). No difference was reported in 24 h sleep duration assessed at six [23] and nine [42,46] months-of-age among infants introduced to complementary foods at ≤12 weeks (around 3 months) compared to at >12 weeks-of-age, at <4 months compared to at *≥*4 months-of-age, and at <26 weeks (at 6 months) compared to at *≥*26 weeks-of-age. However, infants slept 24 min less (−0.39, 95% CI: −0.67–−0.11) at 12 months-of-age if they were introduced to complementary foods at <4 months compared to at *≥*4 months-of-age [23]. 

Only two out of the four studies [42,46], examined the relationship between the timing of introduction to complementary foods and sleep among breastfed infants separately from formula-fed infants. Heinig et al. [42] reported similar results in both breastfed and formula-fed infants, where no difference in 24 h sleep duration was reported in relation to the timing of introduction to complementary foods. However, Morgan et al. [46] found that breastfed infants were more likely to sleep through the night at 9 months with early introduction of complementary foods at ≤12 weeks as compared to at >12 weeks. This was not observed in formula-fed infants. However, the authors were unable to further their investigation due to the lack of data collected on the reasons for infants waking (i.e., waking for feeding or waking and self-soothing to sleep).

#### 3.2.2. Night-Waking Frequency 

One study of infants aged 6–12 months reported no significant association with night-waking frequency and timing of introduction to complementary foods (mean age of introduction 21 weeks); however, an association between later introduction to complementary foods and number of feeds during the night was reported (independent of infant age) [19]. 

### 3.3. Quality Assessment 

All 21 studies were included in the quality assessment (Table 2). Quality scores ranged from 2 to 6 points (out of a total of 6 points). The mean quality score was 4. The quality assessment variable with the lowest score was the additional question on the sleep assessment tool validation, where only 10 out of 21 studies reported using validated sleep assessment methods [17,29,33,34,35,37,40,43,44,45]. Four (19%) studies [17,35,37,44] had a quality score of 6, whilst 14 (66%) [19,23,29,30,31,32,34,38,39,40,41,42,45,46] scored > 4 points, and 3 studies (14%) [33,36,43] scored ≤ 3 points. The three studies had low rating (≤3 points) as their participants were not representative of the target population [33,36,43], had inappropriate outcome measurements [36], had less than 90% completed data collection [33,43], did not account for confounders [33], the feeding group was not administered as intended [43], and did not use a validated sleep assessment tool [36].

## 4. Discussion

The main purpose of this systematic review was to examine the relationship between type of milk feeding, timing of introduction to complementary foods, and sleep in infants aged 12 months and younger. Among infants aged 6 months and younger, a majority of the studies (six out of nine studies, 67%) reported no association between type of milk feeding and 24 h sleep duration. However, studies that examined infant sleep after 6 months-of-age (two out of three studies, 67%) had a greater tendency to report less 24 h sleep duration among breastfed infants compared to formula-fed infants. 

Among infants aged 6 months and younger, no significant difference in night-time sleep duration between different feeding types was reported by half of the studies included in the qualitative synthesis (five out of ten studies, 50%), with two out of three (67%) comparing exclusive breastfeeding to formula-feeding. In contrast, all studies that examined infant sleep after 6 months-of-age reported that breastfeeding was associated with less night-time sleep duration compared to formula-fed infants (two out of two studies), though the total number of studies was limited. Two studies [33,43] classified as low-quality studies had quality ratings of 3 points and were excluded for sensitivity analysis. Removing these two studies from the qualitative synthesis did not change the overall conclusions on the relationship between type of milk feeding and night-time sleep. 

Half (three out of six studies, 50%) of the included studies reported formula-fed infants woke less often than exclusively breastfed infants aged 6 months and younger. This association could be explained by differences in rates of digestion of milk types (i.e., breast milk versus formula), contributing to shorter periods of satiety and a greater number of wakings [48,49]. Another mechanism proposed by Wolke et al., suggests differences in the number of sound signals created by breastfed infants versus formula-fed infants when awake may result in a greater number of identified and reported night wakings in breastfed infants [50]. In addition, breastfeeding mothers have been reported to have an increased sensitivity towards their infant’s cry compared to formula-feeding mothers [51], potentially impacting the number of reported night wakings. In comparison, in five out of seven studies (70%) reporting on breastfed versus formula-fed infants, no differences in the number of night wakings in breastfed infants 6 months-of-age and younger were found compared to formula-fed infants. The similarities in the number of night wakings could be attributed to the mixed consumption of formula and breast milk for both the breastfed and formula-fed infants. Three studies [33,36,43] that reported on night-waking frequency had a low-quality rating (<3 points), and removal of these studies from the qualitative synthesis strengthened the trend of more night wakings observed in exclusively breastfed infants, and no difference in night wakings was observed in breastfed infants in relation to formula-fed infants. These results should be interpreted with caution and should not be used as a basis to change feeding practices, as the WHO recommends exclusive breastfeeding for the first six months of life as best practice for infants [2]. No clear conclusion could be made for type of milk feeding and frequency of night wakings after six months-of-age. 

According to the qualitative synthesis, no associations were reported with the timing of introduction to complementary foods and sleep of infants younger than 12 months. Only one study [42] compared the introduction to complementary foods before and after 26 weeks (i.e., at 6 months-of-age), in accordance with WHO recommendations [2]. No longitudinal study has been carried out to assess infants’ sleep before the introduction of complementary foods through to after the complementary feeding period in their first year of life. Conducting a longitudinal study is the most robust way of addressing the effects of timing of introduction to complementary foods defined by the WHO (<6 months vs 6 months) [2] on sleep outcomes in later infancy and would be an important consideration for future research. The longitudinal design would also allow continuous monitoring of other important aspects of complementary feeding such as the volume and frequency of foods consumed in relation to infant sleep patterns. 

### Strength and Limitations 

Findings from this systematic review are strengthened by the use of a comprehensive search strategy to capture all relevant studies in an extended database. Inclusion and exclusion criteria were well-defined in an effort to remove all studies that were not suited for this review. The use of a comprehensive search strategy, robust inclusion and exclusion criteria, assessment of study quality, and the absence of duplicate trial publications reduced the likelihood of publication bias. Half (6 out of 12) of the included studies that examined the association between feeding type and 24 h sleep duration and total night-time sleep, and more than half (8 out of 15) of the studies that examined the association between night-waking frequency and feeding type, reported no significant differences between milk feeding types, minimizing citation bias in this review. 

However, there are several limitations. Firstly, there was significant heterogeneity in the type of assessment tools used for measuring infant sleep. Some studies only used subjective methods such as retrospective sleep questionnaires or sleep diaries [17,19,30,31,32,33,34,35,36,37,38,39,41,42,46], whilst other studies used objective measures such as an actigraph to capture real-time sleep data [23,29,40,43,44,45]. In addition, the quality of the assessment tool varied, as not all assessment tools were validated, with half (50%) of the studies assessing sleep using non-validated, subjective assessment methods [19,23,30,31,32,36,38,39,41,42,46]. Furthermore, <30% of the included studies used both objective and subjective sleep assessment tools [23,29,40,43,44,45]. Subjective sleep assessment tools are based on parental report, which increases reporting bias [52,53,54] through overestimating total sleep duration (i.e., placement in bed and time of rising) rather than true sleep and wake times determined by real-time measurements such as actigraphs. In addition, parental report may underestimate the number of night wakings [7] compared to actigraph-detected night wakings [54,55,56], as reported wakings are often associated with infant signals such as crying or calling for attention, rather than infant waking that is not associated with any sound [53,54]. Therefore, objective assessments such as actigraph measurements provide a more accurate estimate of sleep variables such as duration, sleep/wake time, and frequency of waking, especially when used in conjunction with a subjective method such as a sleep diary [52,57]. 

Secondly, due to a limited number of included studies and inconsistent data reporting, we were unable to determine the effects of timing of introduction to complementary foods on infant sleep and the effects of type of milk feeding on sleep outcomes such as longest sleep period, sleep onset latency, and duration of night wakings. The lack of attention received for these sleep measures has also been reported by Dias et al. [7]. More studies are required to examine these sleep variables, especially sleep onset latency, a possible indicator of sleep quality [58]. 

Reported age of introduction to complementary foods varied widely across studies (mean age 21 weeks; ≤12 weeks/>12 weeks; <26 weeks/≥ 26 weeks; <4 months/≥ 4 months), with no standardized definitions for the collection of these data [19,23,42,46]. This heterogeneity made comparisons between studies challenging. Furthermore, conclusions from studies that examined the effects of early introduction of complementary foods (i.e., <4 months-of-age) [23,46] or studies that examined the association between late introduction of solids (i.e., ≥7 months) [19] must be interpreted with caution, as this does not align with current recommendations by the WHO [2].

Additionally, of the studies that examined infant milk feeding with and without the addition of complementary foods, none conducted separate analyses according to milk-feeding type with and without complementary food. These studies include infants 6 months and older, with no study examining the association between the volume and frequency of complementary foods consumed in relation to sleep outcomes. This could be a potential confounder since the addition of complementary foods and increase in the volume of food consumed between 6 to 12 months-of-age may influence infant sleep patterns. 

Definitions of night-time and type of milk feeding were not well defined. The window of time considered ‘night-time’ varied, making the comparison of night-time sleep variables challenging. This lack of standardization of variables could account for the diverse range of sleep outcomes. Furthermore, some included studies did not clearly state whether the definition of breastfeeding included exclusive breastfeeding only, or a mixture of exclusively and partially breastfed infants, or infants that were predominantly breastfed. Though some studies have stated breastfeeding as ‘exclusive” or “predominant’, not all defined the terms are in accordance with the WHO definition, in which exclusive breastfeeding for the first 6 months refers to breast milk only with the exception of oral rehydration salts (ORS), drops, and syrups (vitamins, minerals, medicines) [27], while predominant breastfeeding includes the addition of liquids such as water and water-based drinks and fruit juice [27]. For example, Mindell et al. [17] and Huang et al. [39] have defined exclusive breastfeeding as breastmilk only with or without the inclusion of complementary foods among infants aged 3–12 months and 0–8 months, respectively. Therefore, sleep data reported after 6 months-of-age should be interpreted with caution, as complementary foods could impact sleep patterns.

Furthermore, in the qualitative synthesis for type of milk feeding and sleep, one longitudinal [43] and two cohort studies [38,39] assessed sleep at multiple time points, and had differing night-time sleep ranges, night-waking frequency, and longest sleep period outcomes at the different time points. An example of this is reported in the longitudinal study by Rudzik et al., where no association between type of milk feeding and night-time sleep was reported at most time points (4, 6, 8, 10, 12, 16, and 18 weeks-of-age), with an association between breastfeeding and shorter sleep duration at 14 weeks-of-age [43]. 

Finally, most of the included studies were from western countries (North America and Europe), where there are variations in cultural practices associated with infant feeding compared to non-western countries. The inclusion of only English papers is a selection bias that could have contributed to the lack of cultural diversity in the studies included. Therefore, the data should be interpreted with caution and not applied to non-western populations. In addition, not all published studies were included in this review due to our strict inclusion and exclusion criteria. Criteria were strict in an effort to control for heterogeneity in the data. Therefore, the quality of the sleep data are limited to the quality of the individual studies included [59]. 

## 5. Conclusions

This is the first systematic literature review to compare the effects of type of milk feeding on selected sleep variables in infants under 12 months-of-age. Exclusively breastfed infants (≤6 months-of-age) were reported more likely to wake at night compared to formula-fed infants, though this association was not found in breastfed infants (partial or predominantly breastfed). The majority of the studies reported no difference in night-time sleep duration and total 24 h sleep duration in both exclusively breastfed and breastfed infants (≤6 months-of-age) compared to formula-fed infants. However, after 6 months-of-age, most studies reported breastfed infants to sleep less than formula-fed infants. Though studies were limited, the majority observed no association on the timing of introduction to complementary foods and total 24 h sleep duration, including one study that compared infants who were introduced complementary foods before and after 6 months-of-age in accordance with the WHO recommendations.

Further research should evaluate sleep variables such as longest sleep period and sleep onset latency, and most importantly, the effects of the timing of introduction to complementary food on sleep. There is a need for standardized, higher-quality sleep studies in this age group to address the heterogeneity in the type of assessment tool used, the definitions for type of milk feeding and night-time, and limited use of validated and objective tools for sleep assessment. This would provide a better understanding of the relationship between infant feeding and sleep. In addition, to truly understand the complexity between infant feeding (type of milk feeding and timing of introduction to complementary foods) and infant sleep, future research should adopt a longitudinal design, capturing sleep at transition time-points before and after changing feeding methods. 

## Figures and Tables

**Table 1:** Characteristics of studies included in the systematic review by study details, types of sleep variables, type of milk feeding, age of introduction to complementary foods, types of sleep assessment methods, and quality rating (*n* = 22).

Author, Year, Country	Sample Size	Design	Sleep Variables	Type of Milk Feeding ^1^	Age of Introduction to Complementary Foods ^1^	Infant Sleep Assessment ^1^	Quality Rating ^2^
Berger et al., 2017 (United States) [37]	57	Prospective cohort	24 h sleep duration Night-time sleep Night-waking frequency	BF FF	X	BISQ	6
Brown et al., 2015 (United Kingdom) [19]	756	Cross-sectional	Night-waking frequency	BF +/− food FF +/− food	8 to 32 weeks (mean 21.2 weeks)	Questionnaire	5
Butte et al., 1992 (United States) [29]	20	Cross-sectional	Sleep diary: 24 h sleep duration Night-time sleep EEG: Night-waking frequency Sleep onset latency	BF FF	X	5 days sleep diary 1 night ^3^ EEG	5
DeLeon et al., 2007 (United States) [30]	41	Cross-sectional	Duration of night wakings Night-waking frequency Night-time sleep	BF +/− food FF +/− food	X	Questionnaire and sleep diary	4
Figueiredo et al., 2017 (Portugal) [38]	148, 2 weeks 162, 3 months 123, 6 months	Prospective cohort	Night-time sleep Night-waking frequency Duration of night wakings Sleep onset latency Longest sleep period 24 h sleep duration	EBF BF FF	X	24 h infant sleep chronogram	5
Heinig et al., 1993 (United States) [42]	105	Longitudinal study	24 h sleep duration	BF +/− food FF +/− food	<26 weeks ≥26 weeks	Sleep record	4
Huang et al., 2016 (China) [39]	524	Prospective cohort	24 h sleep % Night-waking frequency night-time sleep % Longest sleep period	BF +/− food FF +/− food	X	3 and 7 days Sleep diary ^4^	4
Kaley et al., 2012 (United Kingdom) [31]	74	Cross-sectional	Night-waking frequency Night-time sleep 24 h sleep duration	BF FF	X	3–7 days sleep diary	4
Lee et al., 2000 (South Korea) [32]	188	Cross-sectional	24 h sleep duration Longest sleep period ^5^	EBF FF	X	Sleep diary	4
Mindell et al., 2012 (United States) [17]	92	Prospective Longitudinal	Night-waking frequency Longest sleep period Night-time sleep Sleep onset latency	BF +/− food FF +/− food	X	Expanded BISQ	6
Morgan et al., 2004 (United Kingdom) [46]	1187, type of milk feeding ^6^ 1196, introduction to complementary foods ^6^	Combined RCT	24 h sleep duration	BF FF	≤12 weeks >12 weeks	Questionnaire	5
Nevarez et al., 2010 (United States) [23]	1676, 6 months 1228, 12 months	Prospective cohort	24 h sleep duration	BF +/− food FF +/− food	<4 months > 4 months	1 night EEG Sleep questionnaire	5
Pennestri et al., 2018 (Canada) [41]	388, 6 months 369, 12 months	Cohort study	Sleep through the night—6 h criterion Sleep through the night—8 h criterion	BF +/− food FF +/− food	X	Questionnaire	4
Quillin et al., 1997 (United States) [33]	45	Cross-sectional	Night-time sleep Night-waking frequency	BF FF	X	6 days sleep activity record	3
Quillin et al., 2004 (United States) [34]	33	Cross-sectional	24 h sleep duration Night-time sleep Night-waking frequency ^7^	BF FF	X	5 days sleep diary	4
Rudzik et al., 2018 (United Kingdom) [43]	61	Longitudinal	Night-time sleep Longest sleep period Night-waking frequency	EBF FF	X	1 night actigraph ^8^ and sleep diary	3
Sun et al., 2018 (China) [35]	590	Cross-sectional	Night-waking frequency	BF +/− food FF +/− food	X	Chinese BISQ	6
Tikotzky et al., 2011 (Israel) [44]	56	Longitudinal observational	Night-waking frequency 24 h sleep duration	EBF BF FF	X	4 days actigraph and sleep diary	6
Tikotzky et al., 2015 (Israel) [40]	53	Cohort	Night-waking frequency ^9^ Night-time sleep ^9^	EBF BF FF	X	5 days actigraph and sleep diary, BISQ	5
Wailoo et al., 1990 (United Kingdom) [36]	87	Cross-sectional	Night-waking frequency Longest sleep period	BF FF	X	1 night sleep diary	2
Yoshida et al., 2015 (Japan) [45]	27	Longitudinal	Sleep through the night—6 h criterion Duration of night wakings	EBF FF	X	2 days actigraph 2 days sleep timetable	5

^1^ Abbreviations: BISQ, brief infant sleep questionnaire [47]; EBF, exclusive breastfeeding (review defined); BF, breastfeeding (review defined); FF, formula feeding (review defined); X = no data. ^2^ Mixed methods appraisal tool (MMAT, v.2018) used as the quality assessment tool. ^3^ Definition of one night: 19:00–06:00 h. ^4^ Day and night were defined on a 12 h block of time (8 a.m. to 8 p.m. and 8 p.m. to 8 a.m.). ^5^ Defined as longest sleep between 18:00 and 06:00. ^6^ Only term infants were included. ^7^ Awakenings during mothers’ preferred sleep time. ^8^ Definition of one night: 18:00–08:00 h. ^9^ Raw data provided by the study corresponding author, not found in the published manuscript.

**Table 2:** Type of milk feeding among infants aged ≤ 6 months, >6 months, and 0–12 months in relation to sleep variables including 24 h sleep duration, total night-time sleep, night-waking frequency, duration of night wakings, longest sleep period, and sleep onset latency.

24 h Sleep Duration							
Author, Year	Infant Age at Assessment/Assessment Frequency	≤6 Months		>6 Months	0–12 Months	Statistics ^1^	Quality Rating ^2^
		EBF vs FF ^1^	BF vs. FF ^1^	BF vs. FF ^1^	BF vs. FF ^1^		
Berger et al., 2017 [37]	16 weeks		BF vs. FF mean ± SE (h) 12.95 ± 0.51 vs. 11.43 ± 0.53, *p* = 0.047			*t*-test	6
Butte et al., 1992 [29]	17 weeks		BF vs. FF mean ± SD (h) 13.2 ± 2.3 vs.13.3 ± 0.9, *p* > 0.05			*t*-test Regression	5
Figueiredo et al., 2017 [38]	2, 13, 26 weeks	EBF vs. FF mean ± SD (h) 2 weeks 13.43 ± 2.34 vs. 12.29 ± 2.27, *p* > 0.05 13 weeks 13.05 ± 1.87 vs. 12.87 ± 2.44, *p* > 0.05 26 weeks 12.37 ± 1.76 vs. 12.79 ± 1.05, *p* > 0.05	BF vs. FF mean ± SD (h) 2 weeks 12.18 ± 3.02 vs. 12.29 ± 2.27, *p* > 0.05 13 weeks 12.41 ± 2.21 vs. 12.87 ± 2.44, *p* > 0.05 26 weeks 12.73 ± 1.48 vs. 12.79 ± 1.05, *p* > 0.05			Multivariate Analyses of Chi MANCOVA ^3^	5
Kaley et al., 2012 [31]	4–10 weeks		BF vs. FF Total sleep not assoc. with feeding, *p* > 0.05			Correlation ANOVA	4
Lee et al., 2000 [32]	2–17 weeks	EBF vs. FF mean ± SD (min) 902.4 ± 119.1 vs. 854.8 ± 130.7, *p* < 0.01				Unpaired *t*-test	4
Quillin et al., 2004 [34]	4 weeks		*BF* vs. *FF* mean ± SD (h) 13.1 ± 1.4 vs. 14.4 ± 1.1, *p* = 0.006			*t*-test	4
Tikotzky et al., 2011 [44]	26 weeks	EBF vs. FF No assoc. between total sleep and EBF (r = 0.15, *p* > 0.05)				Spearman rho correlations	6
Nevarez et al., 2010 [23]	26, 52 weeks ^4^		BF +/− food vs. FF +/− food Bivariate 26 weeks β = 0.05 (95%CI: −0.14 to 0.24), *p* > 0.05 Multivariate 26 weeks β = −0.15 (95%CI: −0.37 to 0.07), *p* > 0.05	BF +/− food vs. FF +/− food Bivariate 52 weeks β = 0.02 (95%CI: −0.17 to 0.20), *p* > 0.05 Multivariate 52 weeks is β = −0.17 (95%CI: −0.37 to 0.03), *p* > 0.05		Bivariate Multivariate linear regression ^5^	5
Heinig et al., 1993 [42]	39 weeks			BF +/− food vs. FF +/− food 24 h sleep at 39 weeks greater in FF compared to BF grps, *p* < 0.05		*t*-test	4
Morgan et al., 2004 [46]	39 weeks ^4^			BF +/− food vs. FF +/− food mean ± SE (h) 11.2 ± 0.1 vs. 11.4 ± 0.6, *p* = 0.01 ^6^		ANCOVA ^7^	5
Huang et al., 2016 [39]	0–34 weeks				BF +/− food vs. FF +/− food BF 2.1% lower (30 min less) 24 h sleep % than FF, *p* = 0.0009	Multilevel mixed models	4
**Total Night-Time Sleep**							
**Author, Year**	**Infant Age at Assessment/Assessment Frequency**	**≤6 Months**		**>6 Months**	**0–12 Months**	**Statistics ^1^**	**Quality Rating ^2^**
		**EBF vs. FF ^1^**	**BF vs. FF ^1^**	**BF vs. FF ^1^**	**BF vs. FF ^1^**		
Berger et al., 2017 [37]	16 weeks		BF vs. FF mean ± SE (h) 9.50 ± 0.38 vs. 7.33 ± 0.39, *p* < 0.0001			*t*-test	6
Butte et al., 1992 [29]	17 weeks		BF vs. FF mean ± SD (h) 8.2 ± 1.6 vs. 9.9 ± 1.4, *p* < 0.04			*t*-test Regression	5
Figueiredo et al., 2017 [38]	2, 13, 26 weeks	EBF vs. FF mean ± SD (h) 2 weeks 7.08 ± 1.33 vs. 6.34 ± 1.21, *p* > 0.05 13 weeks 8.06 ± 1.30 vs. 8.27 ± 1.35, *p* > 0.05 26 weeks 8.29 ± 1.36 vs. 8.29 ± 1.07, *p* > 0.05	BF vs. FF mean ± SD (h) 2 weeks 6.77 ± 1.55 vs. 6.34 ± 1.21, *p* > 0.05 13 weeks 8.12 ± 1.22 vs. 8.27 ± 1.35, *p* > 0.05 26 weeks 8.93 ± 1.21 vs. 8.29 ± 1.07, *p* > 0.05			Multivariate Analyses of Chi MANCOVA ^3^	5
Kaley et al., 2012 [31]	4–10 weeks		BF vs. FF NTS duration not assoc. with feeding, *p* > 0.05			Correlation ANOVA	4
Quillin et al., 1997 [33]	4 weeks		BF vs. FF BF infants slept less at night than FF infants. F(1,39) = 4.925, *p* < 0.05			ANOVA-two-way analysis of variance	3
Quillin et al., 2004 [34]	4 weeks		BF vs. FF mean ± SD (h) 6.4 ±1.0 vs. 6.4 ± 0.8, *p* > 0.05			*t*-test	4
Rudzik et al., 2018 [43]	4,6,8,10,12,14,16, 18 weeks	EBF vs. FF Actigraph report No difference between grps for NTS at 2, 6, 8, 10, 12, 14, 16, 18 weeks, *p* > 0.05				*t*-test	3
Yoshida et al., 2015 [45]	13, 17 weeks	EBF vs. FF STN (6 h criterion): 33% vs. 67%				Multiple linear regression	5
Pennestri et al., 2018 [41]	26, 52 weeks		BF +/− food vs. FF +/− food BF infants less likely to STN at 26 weeks (χ^2^ = 26.67, *p* < 0.0001) using 6 h criterion BF infants less likely to STN at 6 months (χ^2^ = 31.19, *p* < 0.0001) using 8 h criterion	BF +/− food vs. FF +/− food BF infants less likely to STN at 52 weeks (χ^2^ = 34.96, *p* < 0.0001) using 6 h criterion BF infants less likely to STN at 12 months (χ^2^ = 25.24, *p* < 0.0001) using 8 h criterion		Chi-squared	4
DeLeon et al., 2007 [30]	39 weeks			BF +/− food vs. FF +/− food BF −ve correlated with total NTS (r = −0.42, *p* < 0.01)		Pearson’s correlation coefficient	4
Huang et al., 2016 [39]	0–34 weeks				BF +/− food vs. FF +/− food No assoc. between NTS %, *p* > 0.05	Multilevel mixed models	4
Mindell et al., 2012 [17]	13–52 weeks ^4^				BF +/− food vs. FF +/− food mean ± SD (h) 10.70 ± 1.03 vs. 10.30 ± 1.31, *p* = 0.146	MANCOVA	6
**Night-Waking Frequency**							
**Author, Year**	**Infant Age at Assessment/Assessment Frequency**	**≤6 Months**		**>6 Months**	**0–12 Months**	**Statistics ^1^**	**Quality Rating ^2^**
		**EBF vs. FF ^1^**	**BF vs. FF ^1^**	**BF vs. FF ^1^**	**BF vs. FF ^1^**		
Berger et al., 2017 [37]	16 weeks		BF vs. FF No difference in no. of NW, *p* > 0.05			*t*-test	6
Butte et al., 1992 [29]	17 weeks		BF vs. FF mean ± SD (no.) 2.9 ± 1.8 vs. 2.7 ± 2.0, *p* > 0.05			*t*-test Regression	5
Figueiredo et al., 2017 [38]	2, 13, 26 weeks	EBF vs. FF mean ± SD (no.) 2 weeks 3.02 ± 0.83 vs. 2.96 ± 0.88, *p* > 0.05 13 weeks 2.19 ± 1.07 vs. 1.65 ± 1.17, *p* > 0.05 26 weeks 2.22 ± 1.01 vs. 1.53 ± 0.90, *p* < 0.01	BF vs. FF mean ± SD (no.) 2 weeks 2.63 ± 0.67 vs. 2.96 ± 0.88, *p* > 0.05 13 weeks 2.18 ± 1.36 vs. 1.65 ± 1.17, *p* > 0.05 26 weeks 1.73 ± 0.94 vs. 1.53 ± 0.90, *p* > 0.05			Multivariate Analyses of Chi MANCOVA ^3^	5
Kaley et al., 2012 [31]	4–10 weeks		BF vs. FF BF woke more freq. than FF, *p* < 0.05			Correlation ANOVA	4
Quillin et al., 1997 [33]	4 weeks		BF vs. FF BF infants had more awakenings F(1,39) = 12.231, *p* < 0.01			ANOVA-two-way analysis of variance	3
Quillin et al., 2004 [34]	4 weeks		BF vs. FF Mean ± SD (no.) ^8^ 2.2 ± 0.8 vs. 2.0 ± 0.9, *p* > 0.05			*t*-test	4
Rudzik et al., 2018 [43]	4, 6, 8, 10, 12, 14, 16, 18 weeks	EBF vs. FF Actigraphy report EBF has 2.1 less NW at 16 weeks, *p* = 0.05 No difference between grps for number of NW at 4, 6, 8, 10, 12, 14, 18 weeks				*t*-test	3
Tikotzky et al., 2011 [44]	26 weeks	EBF vs. FF EBF assoc. with more NW (Actigraph) (r = 0.32, *p* < 0.05)				Spearman rho correlations	6
Tikotzky et al., 2015 [40]	26 weeks	EBF vs. FF Mean ± SD (no.) ^9^ 2.53 ± 1.08 vs. 1.48 ± 0.96, *p* < 0.05				Spearman CC	5
Wailoo et al., 1990 [36]	13–17 weeks		BF vs. FF No difference in no. of NW, *p* > 0.05			*t*-test	2
Brown et al., 2015 [19]	26–52 weeks			BF +/− food vs. FF +/− food No difference in total NW F(1711) = 0.931, *p* = 0.335		MANOVA ^10^	5
DeLeon et al., 2007 [30]	39 weeks			BF +/− food vs. FF +/− food BF +ve correlated with NW frequency (r = 0.48, *p* < 0.01)		Pearson’s correlation coefficient	4
Huang et al., 2016 [39]	0–34 weeks				BF +/− food vs. FF +/− food BF no diff as compared to FF for NW, *p* = 0.0700	Multilevel mixed models	4
Mindell et al., 2012 [17]	13–52 weeks ^4^				BF +/− food vs. FF +/− food mean ± SD (no.) 1.63 ± 1.24 vs. 0.94 ± 0.87, *p* = 0.003	MANCOVA	6
Sun et al., 2018 [35]	8–52 weeks				BF +/− food vs. FF +/− food Freq. NW assoc. with BF (v = 0.18, *p* = 0.002)	Chi-squared *t*-test	
**Duration of Night Wakings**							
**Author, Year**	**Infant Age at Assessment/ Assessment Frequency**	**≤6 Months**		**>6 Months**	**0–12 Months**	**Statistics ^1^**	**Quality Rating ^2^**
		**EBF vs. FF ^1^**	**BF vs. FF ^1^**	**BF vs. FF ^1^**	**BF vs. FF ^1^**		
Figueiredo et al., 2017 [38]	2, 13, 26 weeks	EBF vs. FF mean ± SD (h) 2 weeks 3.87 ± 1.13 vs. 4.38 ± 1.18, *p* > 0.05 13 weeks 3.03 ± 1.16 vs. 3.05 ± 1.20, *p* > 0.05 26 weeks 2.86 ± 1.01 vs. 2.87 ± 1.12, *p* > 0.05	BF vs. FF mean ± SD (h) 2 weeks 4.00 ± 1.11 vs. 4.38 ± 1.18, *p* > 0.05 13 weeks 3.00 ± 1.16 vs. 3.05 ± 1.20, *p* > 0.05 26 weeks 2.14 ± 0.90 vs. 2.87 ± 1.12, *p* > 0.05			Multivariate analyses of Chi MANCOVA ^3^	5
Yoshida et al., 2015 [45]	13, 17 weeks	EBF vs. FF EBF +ve correlated with wake time at night, *p* < 0.01				Multiple linear regression	5
DeLeon et al., 2007 [30]	39 weeks			BF +/− food vs. FF +/− food BF +ve correlated with duration of NW (r = 0.33, *p* < 0.05)		Pearson’s correlation coefficient	4
**Longest Sleep Period**							
**Author, Year**	**Infant Age at Assessment/ Assessment Frequency**	**≤6 Months**		**>6 Months**	**0–12 Months**	**Statistics ^1^**	**Quality Rating ^2^**
		**EBF vs. FF ^1^**	**BF vs. FF ^1^**	**BF vs. FF ^1^**	**BF vs. FF ^1^**		
Figueiredo et al., 2017 [38]	2, 13, 26 weeks	EBF vs. FF mean ± SD (h) 2 weeks 3.04 ± 1.00 vs. 2.82 ± 0.90, *p* > 0.05 13 weeks 5.26 ± 2.15 vs. 6.50 ± 2.44, *p* < 0.05 26 weeks 5.38 ± 2.45 vs. 6.76 ± 1.96, *p* < 0.05	BF vs. FF mean ± SD (h) 2 weeks 3.38 ± 1.12 vs. 2.82 ± 0.90, *p* > 0.05 13 weeks 5.74 ± 2.31 vs. 6.50 ± 2.44, *p* > 0.05 26 weeks 6.98 ± 2.58 vs. 6.76 ± 1.96, *p* > 0.05			Multivariate analyses of Chi MANCOVA ^3^	5
Lee et al., 2000 [32]	2–17 weeks	EBF vs. FF mean ± SD (min) ^11^ 239.9 ± 102.7 vs. 274.1 ± 105.3, *p* < 0.01				Unpaired *t*-test	4
Rudzik et al., 2018 [43]	4, 6, 8, 10, 12, 14, 16, 18 weeks	EBF vs. FF Actigraph report EBF has 55 min-longer LSP at 18 weeks, *p* = 0.04 No difference between grps for LSP at 4, 6, 8, 10, 12, 14, 16 weeks				*t*-test	3
Wailoo et al., 1990 [36]	13–17 weeks		BF vs. FF BF infants more likely to disturb parents within 4 h (χ^2^ = 5.9, DF 3, *p* < 0.01)			*t*-test	2
Huang et al., 2016 [39]	0–34 weeks				BF +/− food vs. FF +/− food No assoc. between LSP *p* > 0.05	Multilevel mixed models	4
Mindell et al., 2012 [17]	13–52 weeks ^4^				BF +/− food vs. FF +/− food mean ± SD (h) 7.06 ± 2.73 vs. 7.85 ± 2.75, *p* = 0.249	MANCOVA	6
**Sleep Onset Latency**							
**Author, Year**	**Infant Age at Assessment/Assessment Frequency**	**≤6 Months**		**>6 Months**	**0–12 Months**	**Statistics ^1^**	**Quality Rating ^2^**
		**EBF vs. FF ^1^**	**BF vs. FF ^1^**	**BF vs. FF ^1^**	**BF vs. FF ^1^**		
Butte et al., 1992 [29]	17 weeks		BF vs. FF EEG: mean ± SD (min) 34.3 ± 41.6 vs. 4.0 ± 12.6, *p* < 0.05			*t*-test Regression	5
Figueiredo et al., 2017 [38]	2, 13, 26 weeks	EBF vs. FF mean ± SD (h) 2 weeks 0.33 ± 0.31 vs. 0.48 ± 0.40, *p* > 0.05 13 weeks 0.42 ± 0.45 vs. 0.42 ± 0.52, *p* > 0.05 26 weeks 0.39 ± 0.35 vs. 0.57 ± 0.72, *p* > 0.05	BF vs. FF mean ± SD (h) 2 weeks 0.56 ± 0.75 vs. 0.48 ± 0.40, *p* > 0.05 13 weeks 0.44 ± 0.41 vs. 0.42 ± 0.52, *p* > 0.05 26 weeks 0.51 ± 0.31 vs. 0.57 ± 0.72, *p* > 0.05			Multivariate analyses of Chi MANCOVA ^3^	5
Tikotzky et al., 2011 [44]	26 weeks	EBF vs. FF EBF assoc. with later sleep onset (r = 0.32, *p* < 0.05)				Spearman rho correlations	6
Mindell et al., 2012 [17]	13–52 weeks ^4^				BF +/− food vs. FF +/− food mean ± SD (h) 0.23 ± 0.15 vs. 0.30 ± 0.53, *p* = 0.427	MANCOVA	6

^1^ Abbreviations: EBF, exclusive breastfeeding (review defined); BF, breastfeeding (review defined); FF, formula feeding (review defined); STN, sleep through the night; NTS, night-time sleep; NW, night wakings; LSP, longest sleep period; CC, correlation coefficient; −ve, negative; +ve, positive; MANCOVA, multivariate analysis of covariance; ANCOVA, analysis of covariance; ANOVA, analysis of variance; MANOVA, multiple analysis of variance; X = no data. ^2^ Mixed methods appraisal tool (MMAT, v.2018) used as the quality assessment tool. ^3^ Adjusted for mother’s age and marital status as covariates. ^4^ Only data from infants 12 months or under were included. ^5^ Adjusted for maternal age, parity, education; household income; infant gender and race/ethnicity. ^6^ Results remained significant after adjusting for social code, level of mother’s education, maternal age, birth order, and parental smoking (*p* = 0.04). ^7^ Adjusted for measurement at 12 weeks, weaning behavior (complementary foods ≤ 12 weeks *v* >12 weeks), milk feeding (breast *v* formula), gender, and (for term infants) whether AGA or SGA. ^8^ Awakenings during mothers’ preferred sleep time. ^9^ Raw data provided by the study corresponding author, not found in the published manuscript. ^10^ Controlled for infant age and birth weight and maternal age and education.^11^ Longest sleep between 18:00 and 06:00.

**Table 3:** Timing of introduction to complementary foods in relation to sleep variables including 24 h sleep duration and night-wakings frequency among infants 12 months-of-age and under.

24 h Sleep Duration				
Author, Year	Infant Age at Assessment/Assessment Frequency	Sleep Outcomes on Introduction to Complementary Foods	Statistics ^1^	Quality Rating ^2^
Morgan et al., 2004 [46]	9 months ^3^	≤12 weeks vs. >12 weeks mean ± SE (h) 11.4 ± 0.1 vs. 11.2 ± 0.1, *p* = 0.07 ^4^	ANCOVA ^5^	5
Nevarez et al., 2010 [23]	6, 12 months ^3^	<4 months vs. ≥4 months Bivariate 6 months Β =−0.20 (95%CI: −0.47 to 0.07), *p* > 0.05 12 months Β = −0.38 (95%CI: −0.64 to −0.12), *p* < 0.05 Multivariate 6 months Β = −0.05 (95%CI: −0.35 to 0.24), *p* > 0.05 12 months Is β = −0.039 (95%CI: −0.67 to −0.11), *p* < 0.05	Bivariate Multivariate linear regression ^6^	5
Heinig et al., 1993 [42]	9 months	<26 weeks vs. ≥26 weeks mean± SE (h) BF group:12.2 ± 1.1 vs. 12.1 ± 1.0, *p* > 0.05 FF group: No associations between age of solid food introduction and 24 h sleep duration	Regression	4
**Night-Waking Frequency**				
**Author, Year**	**Infant Age at Assessment/Assessment Frequency**	**Sleep Outcomes on Introduction to Complementary Foods**	**Statistics ^1^**	**Quality Rating ^2^**
Brown et al., (2015) [19]	6–12 months	Outcomes: 8 to 32 weeks (mean 21.2 weeks) No associations between solid food introduction and NW (r = 0.06, *p* = 0.141)	Pearson’s CC	5

^1^ Abbreviations: BF, breastfeeding; FF, formula feeding; NW, night wakings; CC, correlation coefficient; ANCOVA, analysis of covariance. ^2^ mixed methods appraisal tool (MMAT, v.2018) used as the quality assessment tool. ^3^ Only data from infants 12 months or under were included. ^4^ Adjusted again for social code, level of mother’s education, maternal age, birth order, and parental smoking. ^5^ Adjusted for measurement at 12 weeks, weaning behavior (complementary foods ≤ 12 weeks *v* >12 weeks), milk feeding (breast *v* formula), gender, and (for term infants) whether AGA or SGA. ^6^ Adjusted for maternal age, parity, education; household income; infant gender and race/ethnicity.

## Data Availability

Data sharing not applicable.

## Supplementary Materials

The following are available online at https://www.mdpi.com/article/10.3390/nu13114105/s1, Table S1: Electronic database search strategy for systematic review on infant feeding mode and infant sleep. Table S2: Mixed Methods Appraisal Tool.
