# Supplementary material for: Type of Milk Feeding and Introduction to Complementary Foods in Relation to Infant Sleep: A Systematic Review
Source: Nutrients. 2021 Nov 16;13(11):4105. doi: 10.3390/nu13114105 (PMC8625541; doi:10.3390/nu13114105)
Supplement: Supplementary file 1 [file nutrients-13-04105-s001.zip › table s2.pdf]

nutrients MDPI

Table S2. Mixed Methods Appraisal Tool

Author, Year, Country	Design	Scoring						
		3.1	3.2	3.3	3.4	3.5	3.6	Total score:
Berger et al., 2017 (United States) [37]	Prospective cohort	Y	Y	Y	Y	Y	Y	6
Brown et al., 2015 (United Kingdom) [19]	Cross sectional	Y	Y	Y	Y	Y	N	5
Butte et al., 1992 (United States) [29]	Cross sectional	N	Y	Y	Y	Y	Y	5
DeLeon et al., 2007 (United States) [30]	Cross sectional	N	Y	Y	Y	Y	N	4
Figueiredo et al., 2017 (Portugal) [38]	Prospective cohort	Y	Y	Y	Y	Y	N	5
Heinig et al., 1993 (United States) [42]	Longitudinal study	NS	Y	Y	Y	Y	N	4
Huang et al., 2016 (China) [39]	Prospective cohort	Y	Y	N	Y	Y	N	4
Kaley et al., 2012 (United Kingdom) [31]	Cross sectional	Y	Y	N	Y	Y	N	4
Lee et al., 2000 (South Korea) [32]	Cross sectional	Y	N	Y	Y	Y	N	4
Mindell et al., 2012 (United States) [17]	Prospective Longitudinal	Y	N	Y	Y	Y	N	6
Morgan et al., 2004 (United Kingdom) [46]	Combined RCT	Y	Y	Y	Y	Y	N	5
Nevarez et al., 2010 (United States) [23]	Prospective cohort	Y	Y	Y	Y	Y	N	5
Pennestri et al., 2018 (Canada) [41]	Cohort study	Y	Y	N	Y	Y	N	4
Quillin et al., 1997 (United States) [33]	Cross sectional	N	Y	NS	N	Y	Y	3
Quillin et al., 2004 (United States) [34]	Cross sectional	Y	Y	N	N	Y	Y	4

Rudzik et al., 2018 (United Kingdom) [43]	Longitudinal	**N**	**Y**	**N**	**Y**	**N**	**Y**	3
Sun et al., 2018 (China) [35]	Cross sectional	**Y**	**Y**	**Y**	**Y**	**Y**	**Y**	6
Tikotzky et al., 2011 (Israel) [44]	Longitudinal observational	**Y**	**Y**	**Y**	**Y**	**Y**	**Y**	6
Tikotzky et al., 2015 (Israel) [40]	Cohort	**N**	**Y**	**Y**	**Y**	**Y**	**Y**	5
Wailoo et al., 1990 (United Kingdom) [36]	Cross sectional	**NS**	**N**	**Y**	**N**	**Y**	**N**	2
Yoshida et al., 2015 (Japan) [45]	Longitudinal	**Y**	**Y**	**Y**	**N**	**Y**	**Y**	5

Abbreviations: Y, Yes = 1 point; N, No=0 point; NS, Not Sure=0 point.

Criteria

3.1 Are the participant's representative of the target population?

Explanation: Indicators of representativeness include: clear description of the target population and of the sample (inclusion and exclusion criteria), reasons why certain eligible individuals chose not to participate, and any attempts to achieve a sample of participants that represents the target population.

3.2 Are measurements appropriate regarding both the outcome and intervention (or exposure)?

Indicators of appropriate measurements include: the variables are clearly defined and accurately measured; the measurements are justified and appropriate for answering the research question; the measurements reflect what they are supposed to measure; validated and reliability tested measures of the intervention/exposure and outcome of interest are used, or variables are measured using 'gold standard'.

3.3 Are there complete outcome data?

Decided at 90%

3.4 Are the confounders accounted for in the design and analysis?

Confounders are factors that predict both the outcome of interest and the intervention received/exposure at baseline. They can distort the interpretation of findings and need to be considered in the design and analysis of a non-randomized study. Confounding bias is low if there is no confounding expected, or appropriate methods to control for confounders are used (such as stratification, regression, matching, standardization, and inverse probability weighting).

3.5 During the study period, is the intervention administered (or exposure occurred) as intended?

For intervention studies, consider whether the participants were treated in a way that is consistent with the planned intervention. Since the intervention is assigned by researchers, consider whether there was a presence of contamination (e.g., the control group may be indirectly exposed to the intervention) or whether unplanned co-interventions were present in one group (Sterne et al., 2016).

For observational studies, consider whether changes occurred in the exposure status among the participants. If yes, check if these changes are likely to influence the outcome of interest, were adjusted for, or whether unplanned co-exposures were present in one group (Morgan et al., 2017).

3.6 Is the tool for measuring sleep validated?

Mentioned as validated for sleep, using an objective standard such as actigraph or PSG
