# Supplementary material for: Type of Milk Feeding and Introduction to Complementary Foods in Relation to Infant Sleep: A Systematic Review
Source: Nutrients. 2021 Nov 16;13(11):4105. doi: 10.3390/nu13114105 (PMC8625541; doi:10.3390/nu13114105)
Supplement: Supplementary file 1 [file nutrients-13-04105-s001.zip › tanle s1.pdf]

nutrients MDPI

Table S1. Electronic database search strategy for systematic review on infant feeding mode and infant sleep.

MEDLINE and EMBASE (Ovid)	
**Search No.**	**Search Key words/ statements**
1	infant/ or infant, newborn/ or pediatrics/
2	infan* or pediatric* or paediatric*.mp.
3	new-born*.mp.
4	newborn*.mp.
5	new born*.mp.
6	baby.mp.
7	babies.mp.
8	"Child* under one" or "child* under 12 month*".mp.
9	1 or 2 or 3 or 4 or 5 or 6 or 7 or 8
10	sleep/ or sleep latency/ or sleep stages/
11	actigraphy/ or polysomnography/
12	accelerometry/
13	sleep*.mp.
14	actigraph*.mp.
15	sleep diar*.mp.
16	accelerometer*.mp.
17	the brief infant sleep questionnaire.mp.
18	BISQ.mp.
19	polysomnograph*.mp.
20	PSG.mp.
21	wake.mp.
22	waking*.mp.
23	nap*.mp.
24	videosomnograph*.mp.
25	onset latency.mp.
26	night*.mp.
27	10 or 11 or 12 or 13 or 14 or 15 or 16 or 17 or 18 or 19 or 20 or 21 or 22 or 23 or 24 or 25 or 26
28	feeding behavior/ or bottle feeding/ or breast feeding/
29	milk, human/ or infant formula/
30	bottle Feeding/
31	breastfe* or "feeding mode*".mp.
32	breast-fe*.mp.
33	breast fe*.mp.
34	"human milk*" or " breastmilk".mp.
35	bottle feed*.mp.
36	bottle fed.mp.
37	bottle-fe*.mp.
38	"infant formula*".mp.
39	28 or 29 or 30 or 32 or 33 or 34 or 35 or 36 or 37 or 38
40	weaning/
41	Infant Nutritional Physiological Phenomena/
42	diet/ or eating/
43	feeding Methods/
44	introduction adj3 solid*.mp.
45	wean*.mp.

46	complementary feed* or complementary food*.mp.
47	40 or 41 or 42 or 44 or 45 or 46
48	39 or 47
49	9 and 27 and 48
50	limit 49 to editorial
51	limit 49 to meta analysis
52	limit 49 to "review"
53	limit 49 to "systematic review"
54	50 or 51 or 52 or 53
55	49 not 54
**Scopus**	
**Search No.**	**Search key words/statements**
1	TITLE-ABS-KEY ("infan*" OR "pediatric*" OR "paediatric*" OR "new-born*" OR "newborn*" OR "new born*" OR "baby" OR "babies" OR "child* under one" OR "child* under 12 month*")
2	TITLE-ABS-KEY ("sleep*" OR "actigraph*" OR "sleep diar*" OR "accelerometer*" OR "the brief infant sleep questionnaire" OR "BISQ" OR "polysomnograph*" OR "PSG" OR "wake" OR "waking*" OR "nap*" OR "videosomnograph*" OR "onset latency night*")
3	TITLE-ABS-KEY ("breastfe*" OR "feeding mode*" OR "breast-fe*" OR "breast fe*" OR "human milk*" OR "breastmilk" OR "bottle feed*" OR "bottle fed" OR "bottle-fe*" OR "infant formula*")
4	TITLE-ABS-KEY ("introduction adj3 solid*" OR "wean*" OR "complementary feed*" OR "complementary food*))
5	#3 OR #4
6	#1 AND #2 AND #5 AND (EXCLUDE (DOCTYPE , "re")) AND (EXCLUDE (DOCTYPE , "ed"))
**CINAHL**	
**Search No.**	**Search key words/statements**
1	(MH "Infant") OR (MH "Infant, Newborn")
2	AB Infan* OR AB pediatric* OR AB paediatric* OR AB new-born* OR AB newborn* OR AB new born* OR AB baby OR AB babies OR AB child* under one OR AB "child* under 12 month*"
3	(MH "Sleep") OR (MH "Sleep Latency") OR (MH "Sleep Stages") OR (MH "Sleep Hygiene")
4	(MH "Actigraphy") OR (MH "Polysomnography")
5	(MH "Accelerometry")
6	AB "sleep*" OR "actigraph*" OR "sleep diar*" OR "accelerometer*" OR "the brief infant sleep questionnaire" OR "BISQ" OR "polysomnograph*" OR "PSG" OR "wake" OR "waking*" OR "nap*" OR "videosomnograph*" OR "onset latency night*"
7	(MH "Breast Feeding") OR (MH "Infant Feeding Schedules") OR (MH "Infant Feeding, Supplemental") OR (MH "Infant Feeding") OR (MH "Bottle Feeding") OR (MH "Weaning") OR (MH "Infant Nutrition") OR (MH "Infant Nutritional Physiology")
8	(MH "Infant Formula") OR (MH "Infant Food")
9	AB "breastfe*" OR "feeding mode*" OR "breast-fe*" OR "breast fe*" OR "human milk*" OR "breastmilk" OR "bottle feed*" OR "bottle fed" OR "bottle-fe*" OR " infant formula*"
10	(MH "Feeding Methods")
11	(MH "Eating")
12	AB "introduction adj3 solid*" OR "wean*" OR "complementary feed*" OR "complementary food*"
13	S1 OR S2
14	S3 OR S4 OR S5 OR S6
15	S7 OR S8 OR S9
16	S10 OR S11 OR S12
17	S15 OR S16
18	S13 AND S14 AND S17

CENTRAL	
**Search No.**	**Search key words/statements**
1	MeSH descriptor: [Infant] this term only
2	MeSH descriptor: [Infant, Newborn] this term only
3	("infan*" OR "pediatric*" OR "paediatric*" OR "new-born*" OR "newborn*" OR "new born*" OR "baby" OR "babies" OR "child* under one" OR "child* under 12 month*"):ti,ab,kw (Word variations have been searched)
4	MeSH descriptor: [Sleep] this term only
5	MeSH descriptor: [Sleep Latency] this term only
6	MeSH descriptor: [Sleep Stages] this term only
7	MeSH descriptor: [Actigraphy] this term only
8	MeSH descriptor: [Polysomnography] this term only
9	MeSH descriptor: [Accelerometry] this term only
10	("sleep*" OR "actigraph*" OR "sleep diar*" OR "accelerometer*" OR "the brief infant sleep questionnaire" OR "BISQ" OR "polysomnograph*" OR "PSG" OR "wake" OR "waking*" OR "nap*" OR "videosomnograph*" OR "onset latency night*"):ti,ab,kw (Word variations have been searched)
11	MeSH descriptor: [Breast Feeding] this term only
12	MeSH descriptor: [Bottle Feeding] this term only
13	MeSH descriptor: [Feeding Behavior] this term only
14	MeSH descriptor: [Infant Formula] this term only
15	MeSH descriptor: [Milk, Human] this term only
16	("breastfe*" OR ""feeding mode*" OR "breast-fe*" OR "breast fe*" OR "human milk*" OR " breastmilk" OR "bottle feed*" OR "bottle fed" OR "bottle-fe*" OR "infant formula*"):ti,ab,kw (Word variations have been searched)
17	MeSH descriptor: [Infant Nutritional Physiological Phenomena] this term only
18	MeSH descriptor: [Weaning] this term only
19	MeSH descriptor: [Diet] this term only
20	MeSH descriptor: [Eating] this term only
21	MeSH descriptor: [Feeding Methods] this term only
22	("introduction adj3 solid*" OR "wean*" OR "complementary feed*" OR "complementary food*"):ti,ab,kw (Word variations have been searched)
23	#1 OR #2 OR #3
24	#4 OR #5 OR #6 OR #7 OR #8 OR #9 OR #10
25	#11 OR #12 OR #13 OR #14 OR #15 OR #16
26	#17 OR #18 OR #19 OR #20 OR #21 OR #22
27	#25 OR 26
28	#23 AND #24 AND 2
29	Clicked on trials
